# Setting up an academic research laboratory in Canada in 2015

**DOI:** 10.1186/s40697-015-0086-1

**Published:** 2015-12-01

**Authors:** Emmanuelle Cordat

**Affiliations:** Department of Physiology, Membrane Protein Disease Research Group, University of Alberta, Medical Sciences Building, Room 7-34, Edmonton, AB T6G 2H7 Canada

In 2007, after 6 years of postdoctoral training at the University of Toronto, I was extremely fortunate to be offered an Assistant Professor position in the Department of Physiology at the University of Alberta, Edmonton. Transitioning from a postdoctoral training to an independent investigator position is both extraordinarily exciting and terrifying. On one hand, one has a whole career ahead with just imagination and hard work to shape it as wished. On the other hand, the new recruit has suddenly become “the Boss.” Gone are the late evening dinners with lab mates, coffee breaks complaining about failed experiments, and the reassuring feeling of being part of a well-funded research team. Now has come the time to learn how to motivate trainees, to supervise and manage their work, to ensure their projects are making significant progress toward publication, and to solve personnel issues. While new scientists are heavily trained in performing research, they are usually quite inexperienced in managing human resources. In reality, very few investigators will continue to work at the bench after taking a faculty position, and most of the investigator’s time will be dedicated at managing others’ work. Surprisingly, however, this aspect of the work is often regarded as secondary and much less important than grant writing by new investigators. Although partially true (with no research money, there is no personnel to manage), the type of supervision and interaction with trainees truly determines the success of a laboratory and, therefore, should certainly not be regarded as trivial. Teaching is an additional characteristic of the job, for which new investigators frequently have minimal training. Postdoctoral training is research intensive and leaves little, if any, room for gaining formal teaching experience. New recruits may have 1 to 2 years of teaching relief, but eventually, they have to get prepared to stand up and teach in front of 400 undergraduate students. Setting up a research laboratory also means deciphering the subtle and complex mechanics of the University administrative machine. It can be extremely frustrating to waste hours trying to identify the right person in charge of a specific duty. Hiring a part-time senior research technician, who knows how the system works, can be tremendously helpful in setting up a laboratory.

In addition to managing personnel and teaching, a new investigator has to demonstrate an ability to publish and to bring sufficient research money to sustain research in the laboratory. At this stage, mentors play a tremendous role in determining the success of the new investigator. It can be challenging in a new academic environment to find the right mentor, and institutions have various systems in place to facilitate the process. For female scientists, another level of difficulty can appear to find a mentor with values that align with one’s vision of managing both a scientific career and motherhood. I was fortunate to identify fabulous local and external mentors who were not only supportive and committed but also honest enough to share with me when I was going the wrong way. In 2010, I received a New Investigator salary award from the Kidney Research Scientist Core Education and National Training (KRESCENT) Program. Co-funded by the Kidney Foundation of Canada and the Canadian Institutes for Health Research, the KRESCENT Program offers three types of awards (Doctoral, Post-doctoral, and New Investigators) to increase the number of highly skilled scientists working in preventing end-stage renal disease and in new treatments to improve the health of Canadians affected by kidney diseases. With bi-annual meetings bringing together clinicians, nurses, and basic and public health scientists, this program not only provided a renewed sense of being part of a vibrant research community but also inspiring mentors and friends who were key to the researcher I have become.

Acquiring initial operating funds is a huge step to start a research laboratory. In general, new investigators are seen as less competitive if they do not have operating funds in addition to their start-up and, therefore, are less likely to get funded. But without sufficient operating funds (or with only small start-up funds), it is difficult to acquire solid preliminary data necessary to get funding. Fortunately, some funding agencies within the Canadian scientific community recognize the daunting challenges facing new investigators who are competing with established researchers for a limited pool of funding, especially when the success rate for national funding competitions is as low as 15 %. In an effort to help new investigators obtain those crucial initial operating grants, concessions are made for new investigators during the grant review process, comprising up to a 0.5-point increase in their grant score out of a 5-point scale. Further, one can apply to competitions for funds devoted to New Investigators only, which can be a good alternative to acquire initial operating funds for the recruitment of a first student. After receiving New Investigator operating funds from the Sick Kids Foundation, the Banting Research Foundation, and the Kidney Foundation of Canada, I was able to recruit students and staff and generate the critical preliminary data that enabled me to obtain my first 3-year Canadian Institutes of Health Research (CIHR) operating grant in 2010.

Unfortunately, the climate in the research community had started changing at this time. Success rates for national funding competitions were dropping, provincial funding was on the decline, and academic recruitments had slowed down, all of which brought a general sense of depression in our academic institutions. In every department in my Faculty, researchers started struggling to maintain the momentum which had taken them years to generate, studentships gradually became more and more competitive, and most alarmingly, undergraduate students started turning away from graduate studies and academic careers. My last local graduate student defended her MSc in 2012, and since then, all my graduate students have been international students on a study permit. Although this speaks to the international reputation of Canadian academic research institutions, this alarming trend may result in a dramatic shortage of future university teachers and scientists in Canada in the coming years.

For female researchers, with not only the funding clock but also the biological clock ticking, eventually comes the decision to have children. This is a tough and high-risk decision. Bad timing could jeopardize launching of the laboratory, thereby delaying obtaining a first operating grant and therefore having repercussions on an entire career. On the other side, postponing motherhood cannot be indefinite as with age, fertility (and patience!) decreases. Ultimately, an individual decision needs to be taken at the most appropriate time, while being as much prepared as possible for the unexpected (difficult pregnancy, ill child, etc.) and the expected (sleepless nights, days off work due to sick child, etc.). Having a supportive and flexible partner is invaluable at this stage. Importantly, funding agencies recognize and acknowledge leaves during evaluation processes, and to my knowledge, mothers are rarely penalized for publication delays due to pregnancies and raising young children. Academic institutions also protect academics who have delays in their careers, including for child bearing. Despite all these mechanisms in place, one has to be prepared to see productivity (number of publications, patents, etc.) temporarily slowing down. However, upon return to full-time work, the momentum will eventually be regained.

My first CIHR operating grant ended in 2013. With CIHR transitioning from the traditional open operating grant competition to the new Project and Foundation schemes, it has been incredibly difficult to maintain funding and productivity when success rates of competitions for research funds have significantly dropped. Many principal investigators had to decrease the number of highly qualified personnel in their laboratories, usually by losing the most expensive but also the most experienced individuals, resulting in loss of expertise which has and will inevitably continue to translate to decreased productivity. Canadian researchers are committed to create new knowledge and when possible to translate it into improved quality of life and health for Canadians. With an unstable funding situation and short-term funding, investigators are forced to adopt low-risk projects and research strategies that ultimately may provide only low return on investment. In an uncertain research-funding environment, one would rather publish experimental results sooner and in lower impact journals, thereby lowering the risk of being scooped and maximizing the chances to renew short-term funding, rather than build upon findings to increase the impact of the results. But most importantly, in a more and more competitive world, this unstable environment sacrifices the next generation of researchers, who may either choose other career options or go to more attractive institutions outside Canada where recruitment and funding are more stable.

Overall, starting an academic research career is an incredibly exciting time, and various systems are in place to provide support to new investigators. However, unless the funding situation changes in Canada, one has to be prepared to regularly encounter difficult funding situations in this increasingly competitive environment.

